# Proteomic analysis of symbiotic proteins of *Glomus mosseae* and *Amorpha fruticosa*

**DOI:** 10.1038/srep18031

**Published:** 2015-12-10

**Authors:** Fuqiang Song, Dandan Qi, Xuan Liu, Xiangshi Kong, Yang Gao, Zixin Zhou, Qi Wu

**Affiliations:** 1 Heilongjiang University, Harbin, Heilongjiang, China

## Abstract

Arbuscular mycorrhiza fungi (AMF) can colonize the roots of *Amorpha fruticosa*, a perennial leguminous woody shrub, and form arbuscular mycorrhiza (AM). AMF have significant promoting effects on *A. fruticosa* growth as the intensity of fungal colonization increases. Taking AMF-*A. fruticosa* symbionts as the experimental material, gel-free isobaric tags for relative and absolute quantification (iTRAQ) coupled with two-dimensional liquid chromatography-tandem mass spectrometry (LC-MS/MS) were used to investigate the expression of *A. fruticosa* mycorrhizal proteins at the maturation stage. A total of 3,473 proteins were identified, of which 77 showed dramatic changes in their root expression levels; 33 increased, and 44 decreased. We also found nine AMF proteins that were expressed with AMF treatment. The 77 proteins were classified according to function. Plant proteins were assigned into 11 categories: metabolism-related (32%), protein folding and degradation-related (22%), energy-related (10%), protein synthesis-related (8%), stress and defense-related (24%), transcription-related (6%), membrane and transport-related (4%), cellular structure-related (2.5%), signaling transduction-related (11%) and unknown proteins (5%). The results of the study provide a foundation for further investigation of the metabolic characteristics and molecular mechanisms of AM.

Arbuscular mycorrhiza (AM), representing the most widely distributed mutualistic root symbiosis in nature, are the result of long-term evolution between plant and soil fungi. AM fungi (AMF) are obligate symbionts and are recalcitrant to pure culture on synthetic media; they grow only in living plants. Their hereditary variability and heterogeneous characteristics are essential for colonizing a large number of potential host plants. However, different host plants may simultaneously induce the expression of different symbiosis-related genes. AMF are some of the most widespread microorganisms, and they can form symbionts with more than two-thirds of the vascular plants in natural or artificial ecosystems. These plants include important agricultural species, such as wheat, rice, and the model plant *Populus trichocarpa*^1^,^2^. The foundation of mycorrhizal symbiosis is the ability of AMF, using their multicore hyphae, to provide nutrients (especially phosphorous) to host plants that have long-distance illiquidity^3^. New physiological and molecular evidence has shown that, for phosphorus, the mycorrhizal pathway (MP) is operational regardless of plant growth responses. Meanwhile, the contribution of the direct pathway (DP) is decreased, which results in a greater dependence of host plants on the nutrients that AMF provide^4^. AMF can utilize only simple carbon and nitrogen sources from their hosts to complete their life cycles. This may be due to the loss of some enzyme-encoding genes and to macromolecular synthesis defects that have arisen during the long-term evolution of symbiosis with plants^5^,^6^.

AM play a significant role in promoting the growth of host plants, and researchers have increased their efforts to study the interactions between AMF and host plants. Recorbet and colleagues have compared the root proteome responses of *Medicago truncatula* upon colonization with two AM fungi, i.e., *Glomus mosseae* (GM) and *G. intraradices*, using two-dimensional electrophoresis (2-DE)^7^. They found 42 symbiosis proteins; of these, 32 could be confidently identified and retrieved following MS/MS and matching with a database encompassing 21 fungal proteins. To test the mechanisms by which shoots of Cd-treated mycorrhizal plants avoid metal toxicity, Aloui has performed a 2-DE/MALDI-TOF-based comparative proteomic analysis of the *M. truncatula* shoot responses upon mycorrhization and Cd exposure^8^. finding that Cd triggers an opposite response than mycorrhization, which is coupled with an increase in molecular chaperones in the shoots of mycorrhizal plants relative to those that are metal-free. Wang has studied the dynamic changes in maize leaf protein expression profiles under AMF colonization^9^. In that study, the differentially expressed proteins in maize leaves were separated by 2-DE, and the results reveal 21 differentially expressed gel spots in maize leaves. Among them, 8 proteins were successfully identified. With the development of molecular biology techniques, quantitative analysis of the differences in protein expression profiles during the colonization process of pathogenic or symbiotic microorganisms has become possible; these techniques have played a critical role in analyzing pathogenic mechanisms. Isobaric tags for relative and absolute quantification (iTRAQ) represents one of the new and powerful techniques for simultaneous analysis of multiple samples and provide relative quantification of hundreds of proteins. iTRAQ reagents produce high-quality, reproducible results from complex samples, and iTRAQ has thus become widely used. However, iTRAQ-based studies on the symbiotic mechanisms of AMF and host plants have rarely been reported. Our group has studied the symbiotic relationship between plants and fungi at the mRNA level, Zhang xingxing identified 30 symbiosis-related genes expressed in *Amorpha fruticosa* roots colonized by GM at different stages by using mRNA differential-display PCR (DDRT-PCR). The expressed genes were confirmed by reverse Northern blotting. Eleven fragments were sequenced and putatively identified by homologous alignment, and these genes were found to relate to defense and signal transduction^10^. Kong xiangshi also has found 47 symbiosis-related unigenes during AMF treatment by using suppression subtractive hybridization (SSH) and subsequent Gene Ontology (GO) database, BLAST annotation and literature searches to categorize each of the identified genes. Among the expressed genes, those related to plant metabolism and stress and defense show important roles during the symbiotic process of AMF-*A. fruticosa*^11^. Based on our previous work, we have continued to use *A. fruticosa*, a perennial leguminous woody shrub plant, as a host. Using AMF-*A. fruticosa* symbionts as the experimental materials, we used iTRAQ combined with 2-D LC-MS/MS to investigate the expression of *A. fruticosa* mycorrhizal proteins at the maturation stage. The results of the study provide a theoretical basis for the further analysis of the metabolic characteristics and molecular mechanisms of symbiosis between AMF and *A. fruticosa*.

## Results

### AMF Colonization

**Figure 1:**
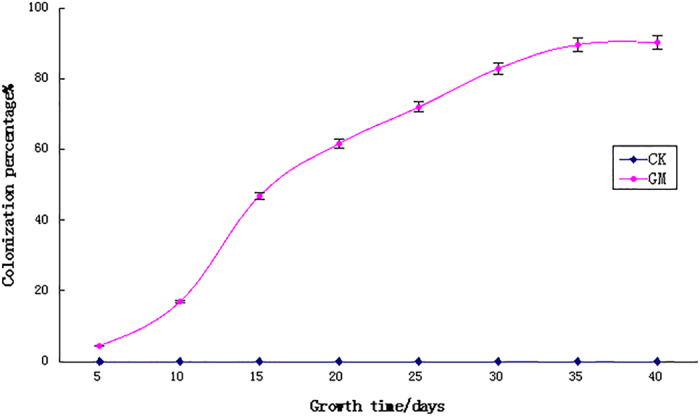
The colonization percentage of *Amorpha fruticosa* roots by AMF changes with time.

The colonization percentage of *A. fruticosa* roots is shown in Fig. 1. At day 5, the roots were relatively small, and only a few hyphae were detected. Most of the spores were not in contact with the host roots and were mainly in their vegetative growth stage^12^,^13^. The infection rate began to increase at day 10, and the growth status of GM-inoculated seedlings was significantly better than that of non-inoculated plants. The colonization percentage increased rapidly at day 15. At that time, there was a large quantity of mycelium-infected roots that increased over time. The colonization percentage reached its peak at the vigorous phase (i.e., 30 days), and a large number of vesicles and arbuscules were observed within the roots. At 40 days, we observed that the hyphae had extended from the plant root surface and had infected neighboring roots. No colonization by AM was observed in the non-inoculated plants, because the mixed soil used for culture had been autoclaved thoroughly.

### Identification of Symbiosis-Related Proteins using iTRAQ LC-MS/MS

The roots of *A. fruticosa* changed their protein levels when colonized by GM, and mutualistic symbionts formed. Using an iTRAQ approach, 86 differentially expressed symbiosis-related proteins were successfully identified. Among them, 77 were plant proteins, with 33 proteins showing increases and 44 showing decreases (Table 1), and 9 were fungal proteins (Table 2). More detailed information in supplementary information. AMF proteins play an import role in symbiotic systems, but they show high expression in only the AMF themselves. The low overall concentration of AMF proteins, and the limitations of the technology, resulted in few AMF proteins being detected^14^.

### Classification of Symbiosis-Related Proteins

**Figure 2:**
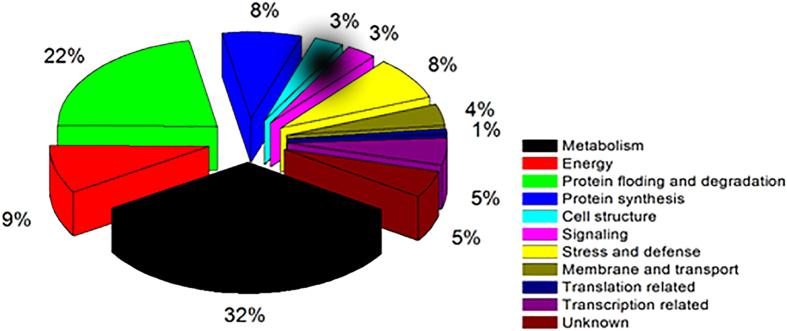
Functional classification of the AM symbiotic-related proteins.

The GO database, BLAST annotations and information reported in the literature were used to categorize each of the identified proteins^11^. The 77 differentially expressed proteins in *A. fruticosa* were categorized into different functional classes and assigned to 11 categories. The functional categories are shown in Fig. 2; they include metabolism-related (32%), protein folding and degradation-related (22%), energy-related (10%), protein synthesis-related (8%), stress and defense-related (24%), transcription-related (6%), membrane and transport-related (4%), cellular structure-related (2.5%), signaling transduction-related (11%) and unknown (5%). Among these classes, proteins related to plant metabolism, protein folding and degradation, and energy (totaling 64% of the identified proteins) play important roles during the symbiotic process of AMF-*A. fruticosa.*

## Discussion

Previous studies have demonstrated that colonization is a multi-step, genetically regulated process under the control of specific loci^15^,^16^. AMF interact with host plants as cell walls, cell membranes and cellular components undergo dramatic changes^17^. During the colonization process, functional proteins are induced to express and regulate this process, ultimately forming stable mutualistic symbionts.

### Signaling-related proteins

Mutualistic symbionts are the result of a mutual recognition and interaction process between AMF and plant signaling molecules. During the colonization process, signal transduction occurs so that the symbiotic partners recognize each other and the host plants decrease their defense responses. At the same time, AMF are prepared to colonize and to form appressoria and, subsequently, to form arbuscules, vesicles and spores. Because of their mutual nutritional relationship, a real-time dynamic signal dialogue between fungi and host plants is continually present. In this study, we found that the protein levels of Rho GDP-dissociation inhibitor 1 and somatic embryogenesis receptor-like kinase were significantly increased in the symbiotic roots.

Rho GDP-dissociation inhibitor 1, a regulator of Rho GTPase, regulates the balance of Rho GTPase bound to GTP or GDP. There are 2 conformational states of Rho GTPase: the GTP-bound ‘active’ state, and the GDP-bound ‘inactive’ state, in which GTP has been hydrolyzed to GDP^18^. As a member of the subfamily of small G proteins, Rho GTPase regulates a number of important signal-transduction pathways in eukaryotic cells. Rho GTPase, called Rop (Rho-related GTPase) in plants, has different isomers in animals and fungi^19^. Rho GTPases are widely distributed in plants, and the corresponding genes in *Arabidopsis*, maize, barley, rice, peas and alfalfa have been cloned^20^,^21^,^22^. Rho GTPases participate in the regulation of a variety of cellular processes, e.g., gene expression, cell wall synthesis, H_2_O_2_ production, actin rearrangement processes, signal transduction pathways of MAP kinase^23^,^24^, and cytoskeletal assembly and reassembly, to produce a variety of cellular responses. As a regulatory factor, RhoGDI1 was significantly increased in *A. fruticosa* AM. Clearly, this protein is closely related to signal transduction between *A. fruticosa* and GM.

Multiple somatic embryogenesis receptor-like kinases (SERKs) have been defined, including the leucine-rich repeat receptor-like kinase (LRR-RLK) subfamily members and a family of transmembrane signal-transduction proteins^25^. They are characterized by a predicted signal sequence, a single transmembrane region, and a cytoplasmic kinase domain. These features suggest that some SERK family protein kinases may play pivotal roles in communication between cells and the environment or in cell-cell interactions. Currently, SERK genes have been cloned from various plant species. The AtSERK3 gene participates in the brassinolide (brassinosteroid, BR) signal-transduction pathway. BR is an important hormone that regulates plant growth and development. Functional analysis has shown that the *Arabidopsis thaliana* mutant became a dwarf when the AtSERK3 gene is knocked out^26^. The overexpression of the OsSERK1 gene in rice cultivars leads to an increase in host resistance to blast fungus^27^; in contrast, transcripts of the lettuce LsSERK gene not only are decreased in *in vitro* somatic embryonic structures but also easily infect *Sclerotinia*^28^. Studies have also shown that the SERK gene is closely related to antibiotic stress. Plant root colonization by AMF results in increased levels of somatic embryogenesis receptor-like kinase, which plays a major role in promoting plant growth and enhancing plant disease resistance.

### Stress and defense-related proteins

Inoculation with AMF has strong growth-promoting effects on *A. fruticosa*, especially at the mature stage of symbiont formation. These effects are mediated by increased action of SERK in BR signal-transduction pathways, which have a key role in the regulation of autoimmune responses and of plant root cell elongation and division. However, such regulation is not determined by a single factor. At an early stage of symbiosis, a weak defense response emerges when roots are stimulated by AMF colonization. Lectin plays a crucial role in this defense response by recognizing and binding to the sugar molecules of intruders and interfering with their function on plants. Many plant lectins can bind to glucose, mannitol, galactose or other monosaccharides, and they exhibit high affinity to the oligosaccharides of alien plants. Studies have shown that lectins on leguminous tree surfaces can gather rhizobia around the roots^29^. As AMF infect the roots of *A. fruticosa*, plant defense responses are initiated, resulting in agglutinin-2 accumulation. Agglutinin-2 is an important factor for the identification of AMF, similarly to rhizobia.

When *A. fruticosa* is colonized by AMF, the abscisic acid (ABA) content increases rapidly, leading to the closing of plant stomata and decreased transpiration; this response also activates the genes encoding soluble osmolytes, thus decreasing stress injuries and the impact of stress-induced reactive oxygen and ethylene^30^. Therefore, ABA accumulation may stimulate metabolic enzymes to produce a feedback effect^31^. The major ABA catabolic route is decomposition via ABA 8′-hydroxylase to form phaseic acid. Therefore, ABA 8′-hydroxylase accumulation in *A. fruticosa* may represent a mechanism for regulating ABA levels.

In multiple rice mapping populations, germin-like protein (GLP) markers have been associated with quantitative trait loci (QTL) for resistance to rice blast pathogens. At the early stage of rice blast fungus infection or mechanical damage, some OsGLPs are transiently induced and expressed. Varying 5′ regulatory regions and the differential expression of some protein family members between resistant and susceptible cultivars correspond with differential hydrogen peroxide (H_2_O_2_) accumulation levels after fungal infection^32^. Wang discovered a new wheat germin-like protein^33^ that is up-regulated in both resistant and susceptible plants. It has been speculated to be involved in wheat defense responses. GLP is significantly increased at the early stage of AMF infection in roots of *A. fruticosa*, and it may participate in biotic stress responses.

### Protein folding and degradation-related proteins

During the symbiosis process, the modification and degradation of peptides and proteins are critical for maintaining cell function. Protein disulfide isomerase, bi-ubiquitin, serine carboxypeptidase, proteasome subunit beta type-6 and subtilisin-like protease SDD1 accumulate in plant roots to ensure proper cell function.

Plants use the proteasome pathway for selective protein degradation, and the proteasome plays pivotal roles in removing abnormally modified proteins and non-targeted proteins. Interactions between bi-ubiquitin and proteasome subunit beta type-6 provide an effective way to degrade proteins. Bi-ubiquitin is highly conserved in eukaryotes, and it is covalently bound to target proteins through post-translational modification to mediate degradation.

Serine carboxypeptidase (SCP), an enzyme that catalyzes the hydrolysis of proteins in eukaryotes, has been found in rice, *Arabidopsis* and peas. It has been shown that SCP has broad functions in plants, including protein turnover and secondary metabolism synthesis, and it plays an important role in improving plant stress resistance. Liu showed that the expression of *OsBISCPL1* was induced by rice blast fungi and antiviral signaling molecules (salicylic acid and jasmonic acid)^34^ and that overexpression of *OsBISCPL1* could enhance disease resistance, oxidative stress tolerance and ABA sensitivity in transgenic *Arabidopsis* plants. *OsBISCPL1* is expressed ubiquitously and differentially in rice, and it is induced by antiviral signaling molecules (BTH, JA, SA and ACC) and is up-regulated by incompatible interactions between rice and the blast fungus. Liu has shown that the expression of the *ZmSCP* gene in corn is up-regulated under induction by *Rhizoctonia solani* and that the ZmSCP protein are associated with various abiotic stresses^35^.

The subtilisin-like protease SDD1 is a member of the processing-type proteases in eukaryotes. As a preproprotein, it can direct peptides for transport to the cytoplasm. SDD1 is a crucial gene that regulates stomatal development and encodes a subtilisin-like serine protease. As a processive enzyme, it may activate a protein molecule or a signal that directs receptors into contact with epidermal cells during stomatal development processes. Liang has shown that the serine protease-encoding gene *SDD1* is widely expressed acts on the development of stomata and is also necessary for normal root development^36^.

Protein disulfide isomerase (PDI), a multifunctional protein, is distributed widely in eukaryotic organisms and is involved in modifying/folding newly synthesized proteins. The catalytic thiol-disulfide exchange reaction to form disulfide is involved in many physiological processes, such as auxiliary protein folding in the endoplasmic reticulum, reconstruction of misfolded proteins, and the repair and refolding of damaged proteins under stress^37^. Additionally, as a chaperone, PDI can assemble heterogeneous protein peptides and regulate disulfide bonds in an ATP-dependent manner, and it may also be closely related to sugar transport, protein synthesis and other metabolic processes in eukaryotic organisms.

### Energy-related Proteins

During the symbiosis process, dihydrolipoyl dehydrogenase, aldehyde dehydrogenase and isocitrate dehydrogenase [NAD] regulatory subunit 1 accumulated in plant roots. AMF colonization significantly enhances the energy metabolism of plants. The Krebs cycle provides more energy than glycolysis, and it is an important pathway not only an important for sugar metabolism but also for the metabolism of lipids, proteins and nucleic acids, which are eventually oxidized to carbon dioxide and water. Isocitrate dehydrogenase (IDH) is considered to be the rate-limiting enzyme of the Krebs cycle; it catalyzes decarboxylation to ketoglutarate while reducing NAD^+^ to NADH^38^. Therefore, the activity of NAD-IDH has a significant impact on cellular metabolism. Isocitrate dehydrogenase [NAD] regulatory subunit 1, a regulatory factor, controls the activity of NAD-IDH and thus affects metabolic activity.

Kuhlemeier has explored the energy metabolism of tobacco pollen and has found that, in vegetative tissues^39^, pyruvate enters the Krebs cycle by pyruvate dehydrogenase (PDH); however, in reproductive organs, it is converted to acetaldehyde by pyruvate decarboxylase (PDC) and then enters the Krebs cycle via aldehyde dehydrogenase (ALDH) and acetyl coenzyme A synthetase (ACS). Thus, ALDH plays an important role in the pyruvate metabolism pathway of PDC/ALDH/ACS. Under stress conditions, plant cells quickly accumulate excessive reactive oxygen species (ROS), which cause oxidative stress and result in the accumulation of large amounts of toxic substance and eventually in plant death^40^. Aldehydes are an important component of peroxidation reaction products, and they play a crucial role in the oxidation of carboxylic aldehydes, the removal of toxic aldehydes, and the reduction of lipid peroxidation, thereby improving plant tolerance^41^. As an important member of the pyruvate dehydrogenase family, dihydrolipoyl dehydrogenases ensure the production of oxidatively decarboxylated pyruvate CoA, and CoA then enters the Krebs cycle to produce large amounts of energy for plant growth.

### Cellular structure-related proteins

Dramatic changes in plant morphology and in the penetrating mycelium, dynamic reorganization of cytoskeletal elements and organelle transformation occur when arbuscular vesicles develop^42^. Tubulin is an important component of the cytoskeleton, and it plays an important role in maintaining intracellular structural order and cell morphology. Meanwhile, tubulin is closely related to cellular transport, cell differentiation, cell motility, signal recognition, cell division and other developmental activities. Mills has revealed dramatic changes in both microtubules and actin arrangement in the host cell, and further studies have found that microtubules and actin rearrangement in the host cell are necessary for expression in non-host plants^43^. Studies on plant tubulin have primarily been focused on annual plants, such as *Arabidopsis*, tobacco, and rice, but study of the tubulin gene in perennial trees has been rare^44^.

### Membrane and transport-related proteins

A K^+^ efflux antiporter and coatomer, which are membrane and transport-related proteins, respectively, were found in the *A. fruticosa* mycorrhizea. The K^+^ efflux antiporter is mainly responsible for maintaining the intracellular ion balance and regulating the cells’ osmotic pressure. During AMF colonization, AMF invasion affects the ion balance of plant root cells, and plants maintain the intracellular ion balance to stimulate K^+^ increases. Coatomer, a coat protein, transports vesicles, and vesicle-mediated non-selective transport ensures the accurate transport of proteins and lipids.

### Metabolism-related proteins

During mycorrhizal symbiosis, increased levels of 3-oxoacyl-[acyl-carrier-protein] synthase, neutral ceramidase and caffeic acid 3-O-methyltransferase (COMT), which are metabolism-related proteins, were observed.

Lipid metabolism is one of the basic metabolic pathways in plants. The β-ketoacyl-acyl carrier protein synthase (KASI)-mediated acyl chain extension is important in the *de novo* synthesis of fatty acids. Ceramides, which are central molecules in the sphingolipid signaling pathway, play important roles as second messengers in plants and participate in many significant plant signaling pathways, such as cell growth, proliferation, differentiation, senescence and apoptosis^45^. Neuraminidase is a key enzyme that regulates ceramide. Neutral neuraminidase hydrolyzes ceramide to form sphingosine (ref). Liu has found that AtCER is involved in H_2_O_2_-induced oxidative stress^46^.

During cell morphogenesis, lignin plays an important role in the growth and development of vascular tissues and is involved in cell wall lignification, which increases the hardness or compressive strength of the cell wall. It also promotes the formation of mechanical tissues while also having a major impact on plant lodging, disease and stress resistance^47^. There are 3 types of monomeric lignin biosynthesis pathways,: the shikimate pathway, the phenylketonuria pathway and the lignin biosynthesis-specific pathway^48^. COMT is a key enzyme in the specific lignin pathway and is involved in the synthesis of S-lignin^49^. AMF colonization enhanced the synthesis of woody *amorpha* lignin, thus affecting the growth and development of plants.

### Transcription and protein synthesis-related proteins

Ribosomal proteins are important components of the ribosome, and they have important roles in translation efficiency and ribosome stability. They also participate in important cellular processes, such as DNA repair, apoptosis and regulation of gene expression; e.g., 40S ribosomal proteins showed significant accumulation in plant roots after AMF invasion.

Nascent polypeptide-associated complex subunit alpha (NAC), which is located at the top of the newly synthesized polypeptide, can reversibly bind to eukaryotic ribosomes and guide the correct distribution and translocation of newly synthesized polypeptides in the cell. The observed increases in 40S ribosomal protein and NAC levels, combined with folding- and degradation-associated proteins, ensure the fast and accurate synthesis and distribution of AM symbiosis-related proteins.

Transcriptional regulation is an important aspect of the regulation of gene expression. The results show significant accumulation of histone H3 and histone H4 in the host plant roots. Nucleosomes constitute the basic unit of chromatin in eukaryotes. Histones, which are structural proteins of chromosomes, play important roles in DNA folding and packing, protecting DNA from digestive enzymes, and gene regulation, tumor formation, and apoptosis. The N-terminal amino acids of histones participate in acetylation, methylation, phosphorylation, ubiquitination and other covalent modifications. Studies have shown that histones may change the structure of chromatin via post-translational modifications, thus modulating gene expression^50^.

### Unknown proteins

During AMF symbiosis, the expression levels of proteins within *A. fruticosa* roots were changed; some proteins disappeared, and new symbiosis proteins arose. The functional analysis of symbiotic proteins in *A. fruticosa*, a non-model plant, is not difficult. Because these proteins were differentially expressed in the symbiotic system, they are targets for future studies.

### AM-A. fruticosa molecular regulation model

**Figure 3:**
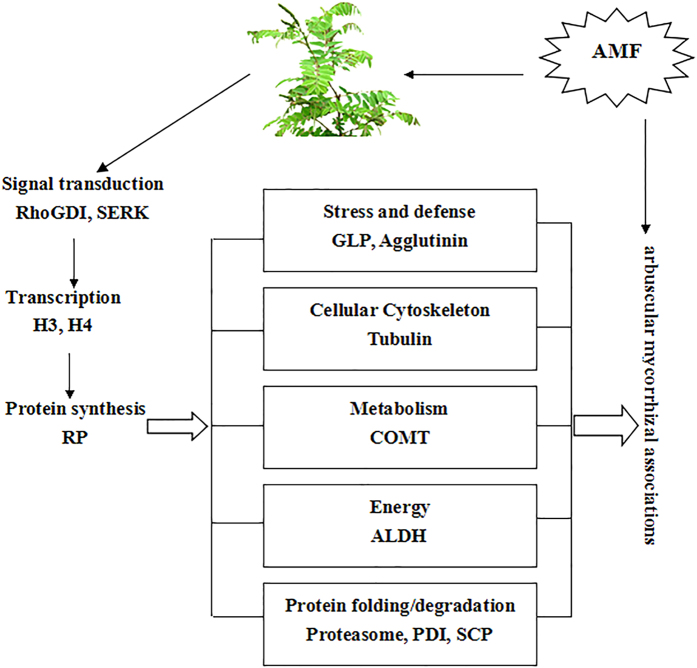
Several biological processes of cellular activities, and it was drawn by dandan Qi.

By using bioinformatics analysis, we found that mycorrhizal proteins were involved in several biological processes and cellular activities (Fig. 3), and we verified that the symbiosis formed between AMF and *A. fruticosa* is a uniform and harmonious result of symbiotic interactions.

## Methods

*G. mosseae* (GM) was harvested from sorghum, which was supplied by the Ecology Laboratory of Heilongjiang University, by co-culturing for longer than 40 days. Inocula contained a mixture of the rhizosphere that consisted of AM fungal spores, hyphae and mycorrhizal fragments. The inocula contained approximately 500 spores per 20 g.

### Seedling culture

Seeds of *A. fruticosa* were purchased from the Academy of Agricultural Sciences of Heilongjiang Province. *A. fruticosa* seeds were sterilized with 0.4% K_2_MnO_4_ for 20 min, rinsed, and then covered with a layer of white gauze to keep them moist. Germination was conducted in an incubator at 30 °C for 60 h after soaking for 24 h. The growing medium was 50% peat soil, 30% vermiculite and 20% sand. It was sterilized in an autoclave at 121°C for 2 h and then air dried for 1 week before the start of the experiments^10^,^11^.

### Mycorrhizal colonization percentage determination

The germinated seeds were then planted in a pre-sterilized mixed matrix and grown under a 16-h photoperiod at temperatures of 25/18 °C (day/night) with 60% relative humidity. One group was inoculated with GM inoculum, and the other was inoculated with sterilized inoculum as a control (CK). Each treatment was repeated 10 times. A total of 20 pots were arranged randomly and watered every 2 days. The mycorrhizal colonization percentage of the seedlings was determined using the Phillip and Hayman staining method (KOH bleaching-acid fuchsin stain) with some modifications (Phillips J M, 1970)^51^.

### Protein extraction, protein quantification and SDS-PAGE

At the maturation stage, *A. fruticosa* roots were harvested, and total root protein was precipitated with 10% (w/v) trichloroacetic acid (TCA) in acetone at −20 °C overnight. After centrifugation at 40,000 × g at 4 °C for 1 h, the pellets were washed 3 times with cold 80% acetone. A 2-D Quant kit (GE Healthcare, USA) was used to determine the protein concentrations. SDS-polyacrylamide gel (12%) electrophoresis was performed with 30-μg samples at 120-V constant voltage for 2 h. The gel was stained with Coomassie blue and visualized^52^,^53^.

### iTRAQ labeling

The CK group and the GM group each included 3 biological replicates. After digesting with trypsin, the proteins from the non-infected and infected samples were labeled with iTRAQ reagents 115 (CK1), 116 (CK2), 117 (CK3), 118 (GM1), 119 (GM2), and 121 (GM3) and were then combined following the manufacturer’s protocol at a ratio of 1:1:1:1:1:1 for LC-MS/MS analysis^54^,^55^.

### LC-MS/MS measurements

The labeled samples were pooled and purified using a strong cation-exchange chromatography (SCX) column and were then separated on an analytical column (1.7 μm, 100 μm × 100 mm) at a flow rate of 300 nL/min using a linear gradient of 5–35% acetonitrile (ACN) over 40 min. The ion spray voltage was 4.5 kV, and nitrogen was used as a nebulizing gas (30 psi) and a curtain gas (15 psi). From each MS scan, the 30 most intense precursor ions were selected for MS/MS fragmentation and were detected at a mass resolution of 30,000 at m/z 400^56^. Data analysis was performed with a Triple TOF 5600 System, and then the iTRAQ data were compared with the protein sequences of homologous species after genome annotation.

### Protein identification

Protein Pilot 4.0 (AB Sciex Inc., USA) was used to simultaneously identify and quantify proteins^57^,^58^. Differentially expressed proteins were required to satisfy 3 conditions for identification: (1) each confident protein identification involved at least 1 unique peptide; (2) the *P*-value was less than 0.05; and (3) changes of greater than 1.2-fold or less than 0.8 fold were considered significant. All of the identified proteins were classified according to the annotations acquired by using the UniProt knowledge base and the GO database.

## Additional Information

**How to cite this article**: Song, F. *et al.* Proteomic analysis of symbiotic proteins of *Glomus mosseae* and *Amorpha fruticosa*. *Sci. Rep.* **5**, 18031; doi: 10.1038/srep18031 (2015).

## Supplementary Material

Supplementary Information

## Figures and Tables

**Table 1:** Identified symbiosis related proteins in *A. fruticosa* roots colonized by *G. mosseae.*

Accession	Protein name	Plant species	Unused score	FM/control
Metabolism				
356523620	3-oxoacyl-[acyl-carrier-protein] synthase I	*Glycine max*	6	6.88
502118474	chorismate synthase, chloroplastic-like isoform X1	*Cicer arietinum*	2.2	1.96
17026394	UDP-glucose pyrophosphorylase	*Amorpha fruticosa*	52	0.43
502122125	pyruvate kinase, cytosolic isozyme	*Cicer arietinum*	26	0.51
502134148	5-methyltetrahydropteroyltriglutamate--homocysteine methyltransferase	*Cicer arietinum*	24	0.52
356505594	sucrose synthase 2	*Glycine max*	22	0.43
356551144	alpha-1,4 glucan phosphorylase L isozyme	*Glycine max*	22	0.33
357453895	4-hydroxy-3-methylbut-2-en-1-yl diphosphate synthase	*Medicago truncatula*	20	0.51
356557483	carbamoyl-phosphate synthase large chain	*Glycine max*	20	0.53
356576733	zeta-carotene desaturase, chloroplastic/chromoplastic	*Glycine max*	20	0.51
356542858	beta-amylase	*Glycine max*	19	0.27
502081957	UDP-glucuronic acid decarboxylase 6-like isoform X3	*Cicer arietinum*	14	0.67
17063848	4-coumarate:CoA ligase	*Amorpha fruticosa*	14	0.47
356566195	alpha-glucan phosphorylase, Hisozyme	*Glycine max*	13	0.32
356552274	glucose-1-phosphate adenylyltransferase small subunit	*Glycine max*	12	0.49
502151454	probable sucrose-phosphate synthase	*Cicer arietinum*	12	0.54
355481047	Neutral invertase	*Medicago truncatula*	11	0.29
356536154	methylthioribose kinase	*Glycine max*	8.6	0.46
357445053	Leukotriene-A4 hydrolase	*Medicago truncatula*	7.6	0.17
84514155	p-coumaroyl-shikimate 3'-hydroxylase	*Trifolium pratense*	6.7	0.19
502131944	carotenoid 9,10(9',10')-cleavage dioxygenase 1-like isoform X1	*Cicer arietinum*	6.2	0.08
68264915	beta-conglycinin alpha subunit	*Glycine max*	5	0.20
356509826	thromboxane-A synthase	*Glycine max*	5.2	0.78
356549592	caffeic acid 3-O-methyltransferase	*Glycine max*	2.8	3.91
502134043	neutral ceramidase	*Cicer arietinum*	6.3	12.65
Signaling				
357446813	Somatic embryogenesis receptor -like kinase	*Medicago truncatula*	12	2.21
502110137	rho GDP-dissociation inhibitor 1	*Cicer arietinum*	2.4	2.86
Stress and defense				
356523918	agglutinin-2	*Glycine max*	5.8	11.47
356527464	transaldolase	*Glycine max*	17	0.52
310704426	stearoyl-acyl carrier protein desaturase	*Phaseolus lunatus*	5.6	0.54
356518971	pantothenate kinase 2	*Glycine max*	5.9	0.61
502180482	abscisic acid 8'-hydroxylase 3	*Cicer arietinum*	2.4	13.80
357438243	Germin-like protein subfamily 1 member	*Medicago truncatula*	2.4	16.44
Membrance and transport				
356526157	coatomer subunit beta'-2	*Glycine max*	16	1.58
356532026	K(+) efflux antiporter 2, chloroplastic	*Glycine max*	1.5	24.89
75219328	Protein TIC110, chloroplastic	*Pisum sativum (pea)*	10	0.42
Energy				
356521795	dihydrolipoyl dehydrogenase	*Glycine max*	22	3.44
356552735	isocitrate dehydrogenase [NAD] regulatory subunit 1	*Glycine max*	2.2	1.67
502163841	aldehyde dehydrogenase family 2 member C4	*Cicer arietinum*	2.2	3.98
48927683	putative inorganic pyrophosphatase	*Arachis hypogaea*	5.6	0.26
372450305	ATPase subunit 1 (mitochondrion)	*Lotus japonicus*	41	0.30
356520768	stellacyanin	*Glycine max*	2.1	0.43
9280616	NADH dehydrogenase subunit 9	*Lupinus angustifolius*	16	0.38
Protein folding and degradation				
502132065	proteasome subunit beta type-6	*Cicer arietinum*	21	2.34
355518872	Bi-ubiquitin	*Medicago truncatula*	18	2.51
356547865	serine carboxypeptidase II-3	*Glycine max*	8.3	42.54
356500665	serine carboxypeptidase 24-like isoform 2	*Glycine max*	5.7	3.20
356540970	serine carboxypeptidase-like 34	*Glycine max*	5	2.78
163914235	subtilase	*Lotus japonicus*	3	2.72
356514109	subtilisin-like protease SDD1	*Glycine max*	2.1	1.31
502162590	oligopeptidase A	*Cicer arietinum*	17	0.59
49257109	protein disulfide isomerase	*Glycine max*	18	1.28
356548123	26S proteasome regulatory subunit 4 homolog A	*Glycine max*	15	0.55
502142068	26S protease regulatory subunit 8 homolog A	*Cicer arietinum*	28	0.48
356553349	probable 26S proteasome non-ATPase regulatory subunit 3	*Glycine max*	16	0.61
357474441	26S proteasome non-ATPase regulatory subunit	*Medicago truncatula*	26	0.61
502090101	T-complex protein 1 subunit zeta	*Cicer arietinum*	24	0.50
356513012	T-complex protein 1 subunit delta-like isoform 2	*Glycine max*	22	0.44
357493557	T-complex protein 1 subunit eta	*Medicago truncatula*	19	0.58
355515447	Peptidyl-prolyl cis-trans isomerase E	*Medicago truncatula*	6	0.10
Protein synthesis				
356508574	40S ribosomal protein S9–2	*Glycine max*	24	3.34
356521522	40S ribosomal protein S16	*Lupinus polyphyllus*	10	1.87
356571876	Nascent polypeptide-associated complex subunit alpha	*Medicago truncatula*	2.1	1.43
2500521	Eukaryotic initiation factor 4A-15	*Nicotiana tabacum*	34	0.62
356548401	lysyl-tRNA synthetase	*Glycine max*	15	0.48
502168167	methionine aminopeptidase 1A	*Cicer arietinum*	4.1	0.77
Translation related				
194466266	perchloric acid soluble translation inhibitor protein	*Arachis hypogaea*	9.2	3.20
Transcription related				
357479669	Histone H4	*Medicago truncatula*	14	6.36
357485127	Histone H3	*Medicago truncatula*	4.7	2.60
502098976	small nuclear ribonucleoprotein-associated protein B'-like isoform X2	*Cicer arietinum*	5.5	1.92
356547438	pre-mRNA-processing-splicing factor 8	*Glycine max*	31	0.66
Cellular structure				
502138074	tubulin beta-1 chain	*Cicer arietinum*	8.1	6.32
356545743	myosin-Vb	*Glycine max*	3.6	0.77
Unkonw				
291047846	unknown	*Glycine max*	22	0.69
359807666	unknown	*Medicago truncatula*	6	3.37
257688087	unknown	*Glycine max*	5.6	7.47
351725945	unknown	*Glycine max*	4	2.03

**Note**: Unused score represent this data was significant; FM/control represent the changes of more than 1.2 or less than 0.8 fold were considered as significant.

**Table 2:** Fungal proteins expressed in AM identified by iTRAQ approach.

Accession	Protein name	Fungus species	Unused score	FM/control
Protein folding				
90970323	heat shock protein 60	*Rhizophagus intraradices*	19.71	14.5199
76780890	binding protein	*Rhizophagus intraradices*	2.21	16.0671
Cellular structure				
52626570	alpha-tubulin	*Glomus diaphanum*	8.82	18.8814
219553143	beta-tubulin	*Rhizophagus clarus*	13.04	15.417
Metabolism				
378404947	fumarate reductase	*Rhizophagus intraradices*	11.97	7.7834
8134607	Phosphoglycerate kinase	*Funneliformis mosseae*	3.37	25.881
38146200	glutamine synthetase	*Funneliformis mosseae*	3.21	11.6025
Protein synthesis				
82792162	elongation factor 1-alpha, partial	*Scutellospora heterogama*	8.03	6.8289
Energy				
254212205	F-ATPase beta subunit, partial (mitochondrion)	*Glomus custos*	8.03	15.8362

**Note**: Unused score represent this data was significant; FM/control represent the changes of more than 1.2 or less than 0.8 fold were considered as significant.
