# Supplementary material for: Proteomic analysis of symbiotic proteins of Glomus mosseae and Amorpha fruticosa
Source: Sci Rep. 2015 Dec 10;5:18031. doi: 10.1038/srep18031 (PMC4674871; doi:10.1038/srep18031)
Supplement: Supplementary Information [file srep18031-s1.doc]

**Proteomic analysis of symbiotic proteins of *Glomus mosseae* and *Amorpha fruticosa***

^1,*^fuqiang Song, ^1^dandan Qi, ^1^xuan Liu, ^1^xiangshi Kong, ^1^yang Gao, ^1^zixin Zhou, ^1^qi Wu

^1^Heilongjiang University, Harbin, Heilongjiang, China;

^*^Corresponding author (0431sfq@163.com)

**Supplementary dataset 1**

| Accession | Description | Index | Unused | %Cov | %Cov(50) | %Cov(95) | Peptides(95%) | GM1/CK3 | PVal GM1/CK3 | GM2/CK3 | PVal GM2/CK3 | GM3/CK3 | PVal GM3/CK3 |
| --- | --- | --- | --- | --- | --- | --- | --- | --- | --- | --- | --- | --- | --- |
| gi|17026394 | UDP-glucose pyrophosphorylase [Amorpha fruticosa] | 10 | 51.9 | 73.25000167 | 62.00000048 | 61.77999973 | 48 | 0.420726597 | 0.00483177 | 0.469894111 | 0.01488656 | 0.432513803 | 0.0104646 |
| gi|372450305 | ATPase subunit 1 (mitochondrion) [Lotus japonicus] | 22 | 41.35 | 60.19999981 | 48.32000136 | 45.35000026 | 35 | 0.401790798 | 0.042266119 | 0.319153786 | 0.01447843 | 0.2108628 | 0.01107706 |
| gi|257726659 | unnamed protein product [Glycine max] | 35 | 33.79 | 58.60000253 | 47.94000089 | 40.18999934 | 20 | 0.487528503 | 0.01078121 | 0.704693079 | 0.200675994 | 0.597035289 | 0.066873237 |
| gi|148872938 | ATP citrate lyase alpha subunit [Glycyrrhiza uralensis] | 45 | 29.23 | 57.73000121 | 37.34000027 | 31.25 | 16 | 0.405508488 | 0.020089019 | 0.642687678 | 0.074603572 | 0.570164323 | 0.053338729 |
| gi|359806735 | uncharacterized protein LOC100812783 [Glycine max] | 51 | 28.24 | 66.99000001 | 44.49999928 | 42.34000146 | 16 | 0.413047493 | 0.03531491 | 0.501187205 | 0.019470081 | 0.524807513 | 0.036950771 |
| gi|357474441 | 26S proteasome non-ATPase regulatory subunit [Medicago truncatula] | 60 | 26.42 | 35.89000106 | 21.89999968 | 18.17000061 | 15 | 0.666806817 | 0.0376679 | 0.672976673 | 0.09364596 | 0.731139123 | 0.097657621 |
| gi|502156320 | PREDICTED: dihydrolipoyllysine-residue acetyltransferase component 2 of pyruvate dehydrogenase complex, mitochondrial-like [Cicer arietinum] | 65 | 25.09 | 42.77999997 | 27.95999944 | 27.03999877 | 16 | 2.421029091 | 0.046187431 | 1.066596031 | 0.40805009 | 1.976969957 | 0.123676002 |
| gi|502090101 | PREDICTED: T-complex protein 1 subunit zeta-like [Cicer arietinum] | 72 | 24.19 | 44.11000013 | 34.38999951 | 29.71999943 | 14 | 0.515228629 | 0.007899608 | 0.492039502 | 0.02980281 | 0.444631308 | 0.02302934 |
| gi|255629938 | unknown [Glycine max] | 75 | 23.95 | 64.41000104 | 40.09000063 | 36.93999946 | 14 | 3.40408206 | 7.58E-06 | 3.221069098 | 1.53E-05 | 3.28095293 | 7.90E-06 |
| gi|257742399 | unnamed protein product [Glycine max] | 79 | 23.74 | 57.26000071 | 44.11000013 | 44.11000013 | 20 | 0.648634374 | 0.024398999 | 0.704693079 | 0.141065493 | 0.654636085 | 0.067075387 |
| gi|356505594 | PREDICTED: sucrose synthase 2-like [Glycine max] | 95 | 22.29 | 43.09999943 | 28.33000124 | 20.4400003 | 26 | 0.383707315 | 0.028377609 | 0.383707315 | 0.02323759 | 0.469894111 | 0.026991811 |
| gi|356521795 | PREDICTED: dihydrolipoyl dehydrogenase-like [Glycine max] | 97 | 22.15 | 42.60999858 | 27.34000087 | 25.44000149 | 13 | 2.992264986 | 3.01E-06 | 3.46736908 | 1.25E-06 | 3.250873089 | 2.01E-06 |
| gi|356513012 | PREDICTED: T-complex protein 1 subunit delta-like isoform 2 [Glycine max] | 101 | 21.87 | 47.47000039 | 30.77000082 | 25.33000112 | 13 | 0.401790798 | 0.069012448 | 0.608134985 | 0.273636997 | 0.440554887 | 0.0176786 |
| gi|356551144 | PREDICTED: alpha-1,4 glucan phosphorylase L isozyme, chloroplastic/amyloplastic-like [Glycine max] | 102 | 21.86 | 37.00999916 | 18.29999983 | 13.60000074 | 12 | 0.413047493 | 0.052634481 | 0.602559626 | 0.041704722 | 0.331131101 | 0.01357299 |
| gi|291047846 | unnamed protein product [Glycine max] | 105 | 21.55 | 48.87999892 | 29.48000133 | 21.0800007 | 12 | 0.73790431 | 0.09568014 | 0.698232412 | 0.017622549 | 0.779830098 | 0.88145411 |
| gi|388508100 | unknown [Lotus japonicus] | 114 | 21.23 | 69.5299983 | 65.67000151 | 57.08000064 | 19 | 2.606153011 | 0.006805027 | 1.803017974 | 0.056037869 | 2.089296103 | 0.01841869 |
| gi|356557483 | PREDICTED: carbamoyl-phosphate synthase large chain-like [Glycine max] | 127 | 20.24 | 33.75999928 | 15.42000026 | 9.561000019 | 9 | 0.510505021 | 0.002512219 | 0.816582382 | 0.477949798 | 0.505824685 | 0.03151568 |
| gi|356576733 | PREDICTED: zeta-carotene desaturase, chloroplastic/chromoplastic-like [Glycine max] | 132 | 19.95 | 35.6099993 | 23.68000001 | 21.93000019 | 13 | 0.619441092 | 0.152269796 | 0.744732022 | 0.164743498 | 0.505824685 | 0.04174269 |
| gi|90970323 | heat shock protein 60 [Rhizophagus intraradices] | 134 | 19.71 | 44.24000084 | 26.10000074 | 22.54000008 | 11 | 13.80383968 | 3.25E-06 | 14.99685001 | 1.50E-06 | 15.41699982 | 1.48E-06 |
| gi|358248074 | 1-deoxy-D-xylulose 5-phosphate reductoisomerase, chloroplastic-like [Glycine max] | 136 | 19.57 | 36.0799998 | 30.1699996 | 28.69000137 | 13 | 0.299226493 | 0.03942905 | 0.679203629 | 0.196242601 | 0.478630096 | 0.073838674 |
| gi|502098144 | PREDICTED: T-complex protein 1 subunit eta-like [Cicer arietinum] | 146 | 18.91 | 48.3099997 | 19.60999966 | 17.11000055 | 12 | 0.57543987 | 0.02948099 | 0.666806817 | 0.057433002 | 0.597035289 | 0.044205781 |
| gi|356542858 | PREDICTED: beta-amylase-like [Glycine max] | 156 | 18.51 | 31.85000122 | 22.17999995 | 22.17999995 | 21 | 0.461317599 | 0.185222805 | 0.267916799 | 0.03749473 | 0.55462569 | 0.125269398 |
| gi|502115108 | PREDICTED: poly(rC)-binding protein 1-like [Cicer arietinum] | 161 | 18.3 | 28.7800014 | 20.29999942 | 15.12999982 | 9 | 1.737800956 | 0.01437481 | 1.629295945 | 0.021413211 | 1.690441012 | 0.01669299 |
| gi|502162590 | PREDICTED: oligopeptidase A-like [Cicer arietinum] | 194 | 16.91 | 33.03999901 | 20.12999952 | 12.40999997 | 9 | 0.724435985 | 0.034280989 | 0.625172675 | 0.058583379 | 0.816582382 | 0.323899597 |
| gi|356527464 | PREDICTED: transaldolase-like [Glycine max] | 203 | 16.53 | 40.54999948 | 21.87000066 | 20.49999982 | 13 | 0.487528503 | 0.03883015 | 0.564936996 | 0.061210349 | 0.642687678 | 0.097608417 |
| gi|356526157 | PREDICTED: coatomer subunit beta'-2-like [Glycine max] | 215 | 16.25 | 26.9600004 | 12.71000057 | 9.833999723 | 8 | 2.754229069 | 0.002066018 | 1.342764974 | 0.003709432 | 1.584892988 | 0.00292459 |
| gi|9280616 | NADH dehydrogenase subunit 9 [Lupinus angustifolius] | 220 | 16 | 48.42000008 | 45.26000023 | 45.26000023 | 8 | 0.879022479 | 0.686271191 | 0.724435985 | 0.184268206 | 0.380189389 | 0.02991 |
| gi|356553349 | PREDICTED: probable 26S proteasome non-ATPase regulatory subunit 3-like [Glycine max] | 223 | 15.83 | 43.47000122 | 24.07999933 | 18.97999942 | 7 | 0.839460015 | 0.456622213 | 0.549540877 | 0.002459734 | 0.630957425 | 0.009015225 |
| gi|356548401 | PREDICTED: LOW QUALITY PROTEIN: lysyl-tRNA synthetase-like [Glycine max] | 249 | 14.69 | 39.21000063 | 14.8300007 | 10.71000025 | 7 | 0.529663384 | 0.024127319 | 0.452897608 | 0.01256975 | 0.478630096 | 0.01098357 |
| gi|300633911 | unnamed protein product [Amorpha fruticosa] | 265 | 14.39 | 44.44000125 | 22.95999974 | 19.63 | 8 | 0.510505021 | 0.019554211 | 0.55462569 | 0.02689389 | 0.549540877 | 0.080755122 |
| gi|54039313 | RecName: Full=40S ribosomal protein S13 | 274 | 14.07 | 68.87000203 | 48.33999872 | 35.76000035 | 8 | 1.870682001 | 0.049200621 | 2.376840115 | 0.01473117 | 2.398833036 | 0.007877549 |
| gi|357479669 | Histone H4 [Medicago truncatula] | 276 | 14.04 | 28.61000001 | 15.48999995 | 14.16999996 | 23 | 6.606935024 | 0.007466971 | 6.486343861 | 0.005094769 | 7.655965805 | 0.00430672 |
| gi|356557066 | PREDICTED: nuclear-pore anchor-like [Glycine max] | 315 | 13.23 | 24.05000031 | 5.976000056 | 3.27700004 | 7 | 1.570363045 | 0.030202581 | 2.992264986 | 0.0106268 | 2.108628035 | 0.117092803 |
| gi|219553143 | beta-tubulin [Rhizophagus clarus] | 322 | 13.04 | 56.15000129 | 39.59999979 | 35.35000086 | 24 | 19.58844948 | 0.029169319 | 12.8233099 | 0.03089631 | 15.41699982 | 0.02962452 |
| gi|356527232 | PREDICTED: alpha-glucan phosphorylase, H isozyme-like [Glycine max] | 326 | 12.98 | 31.31999969 | 16.67000055 | 15.71999937 | 12 | 0.405508488 | 0.0419764 | 0.192309201 | 0.017430089 | 0.346736789 | 0.046579991 |
| gi|502078783 | PREDICTED: 40S ribosomal protein S5-like [Cicer arietinum] | 373 | 12.12 | 48.3099997 | 20.76999992 | 16.42999947 | 7 | 2.290868044 | 0.009849271 | 1.472311974 | 0.297929287 | 2.051162004 | 0.059979361 |
| gi|378404947 | fumarate reductase [Rhizophagus intraradices] | 383 | 11.97 | 38.94000053 | 19.37000006 | 14.2900005 | 6 | 5.345643997 | 0.02070782 | 7.17794323 | 0.001759428 | 5.495409012 | 0.038708709 |
| gi|257671734 | unnamed protein product [Glycine max] | 389 | 11.92 | 34.36999917 | 20.38999945 | 15.72999954 | 6 | 0.469894111 | 0.0150763 | 0.630957425 | 0.083500691 | 0.452897608 | 0.01389766 |
| gi|502151454 | PREDICTED: probable sucrose-phosphate synthase-like [Cicer arietinum] | 393 | 11.82 | 29.10999954 | 11.81000024 | 6.333000213 | 7 | 0.478630096 | 0.021233501 | 0.529663384 | 0.02261617 | 0.510505021 | 0.01895182 |
| gi|356553146 | PREDICTED: uncharacterized protein LOC100810630 [Glycine max] | 468 | 10.48 | 25.99999905 | 8.032000065 | 6.627000123 | 5 | 0.492039502 | 0.051135551 | 0.469894111 | 0.133358896 | 0.424619585 | 0.035120752 |
| gi|356521522 | PREDICTED: 40S ribosomal protein S16-like [Glycine max] | 482 | 10.33 | 47.44000137 | 33.97000134 | 28.8500011 | 5 | 1.721868992 | 0.01287178 | 2.013724089 | 0.01065812 | 1.870682001 | 0.01126678 |
| gi|356514170 | PREDICTED: uncharacterized protein LOC100801730 [Glycine max] | 517 | 10 | 31.49999976 | 25.08000135 | 24.77000058 | 8 | 2.312064886 | 0.046053842 | 1.202263951 | 0.528776586 | 1.30617094 | 0.300277501 |
| gi|351726002 | uncharacterized protein LOC100500302 [Glycine max] | 561 | 9.24 | 43.09000075 | 28.18999887 | 28.18999887 | 6 | 3.221069098 | 0.030477719 | 3.46736908 | 0.02176901 | 3.564511061 | 0.021516209 |
| gi|52626570 | alpha-tubulin [Glomus diaphanum] | 603 | 8.82 | 42.03000069 | 29.46999967 | 24.63999987 | 9 | 18.53532028 | 0.000386827 | 20.13723946 | 0.000367407 | 19.7696991 | 0.000371514 |
| gi|356536154 | PREDICTED: methylthioribose kinase-like [Glycine max] | 621 | 8.58 | 36.44999862 | 18.23000014 | 10.54999977 | 4 | 0.519995987 | 0.049303051 | 0.380189389 | 0.005503849 | 0.401790798 | 0.0116864 |
| gi|356547865 | PREDICTED: serine carboxypeptidase II-3-like [Glycine max] | 656 | 8.25 | 20.76999992 | 14.04999942 | 8.961000293 | 6 | 59.15615845 | 0.000380092 | 55.97576141 | 0.000470887 | 57.0164299 | 0.000492406 |
| gi|502121018 | PREDICTED: germin-like protein subfamily 3 member 2-like [Cicer arietinum] | 677 | 8.1 | 39.23000097 | 21.52999938 | 17.70000011 | 4 | 1.380383968 | 0.122591503 | 1.940886021 | 0.02996153 | 1.318256974 | 0.276985586 |
| gi|502138074 | PREDICTED: tubulin beta-1 chain-like [Cicer arietinum] | 678 | 8.09 | 61.43000126 | 48.6499995 | 38.33999932 | 27 | 7.244359016 | 0.0117861 | 6.546361923 | 0.01088859 | 7.244359016 | 0.01225225 |
| gi|82792162 | elongation factor 1-alpha, partial [Scutellospora heterogama] | 694 | 8.03 | 46.59999907 | 33.25000107 | 18.19999963 | 14 | 5.970353127 | 0.009939361 | 6.251727104 | 0.008895616 | 5.861382008 | 0.009393292 |
| gi|254212205 | F-ATPase beta subunit, partial (mitochondrion) [Glomus custos] | 695 | 8.03 | 66.18000269 | 55.55999875 | 41.54999852 | 13 | 18.36537933 | 0.01197189 | 21.6770401 | 0.01236875 | 23.76840019 | 0.01045648 |
| gi|357445053 | Leukotriene-A4 hydrolase-like protein [Medicago truncatula] | 740 | 7.61 | 25.69999993 | 13.33999932 | 12.35999987 | 5 | 0.1786488 | 0.048262399 | 0.654636085 | 0.402822614 | 0.544502676 | 0.166857705 |
| gi|357493899 | Vesicle-associated membrane protein 7C [Medicago truncatula] | 774 | 7.35 | 32.87999928 | 21.92000002 | 10.05000025 | 3 | 0.648634374 | 0.174496904 | 0.591561615 | 0.029469309 | 0.390840888 | 0.1100135 |
| gi|84514155 | cytochrome P450 monooxygenase CYP98A37 [Medicago truncatula] | 850 | 6.66 | 29.67000008 | 8.055000007 | 6.876000017 | 3 | 0.963828981 | 0.608526289 | 0.376703799 | 0.047118351 | 0.946237087 | 0.500157118 |
| gi|502134043 | PREDICTED: neutral ceramidase-like [Cicer arietinum] | 917 | 6.29 | 25.87000132 | 9.249000251 | 5.897999927 | 3 | 23.33457947 | 0.001628826 | 26.30267906 | 0.001617228 | 15.55965996 | 0.001753583 |
| gi|502131944 | PREDICTED: carotenoid 9,10(9',10')-cleavage dioxygenase 1-like isoform X1 [Cicer arietinum] | 928 | 6.23 | 25.24999976 | 7.965999842 | 6.949000061 | 6 | 0.307609707 | 0.057316858 | 0.083176367 | 0.00906721 | 0.074473187 | 0.033672519 |
| gi|359807666 | uncharacterized protein LOC100785835 [Glycine max] | 997 | 6.01 | 29.58999872 | 17.98000038 | 17.98000038 | 3 | 2.488857031 | 0.003925099 | 4.875285149 | 0.002780505 | 3.372873068 | 0.003298199 |
| gi|388514621 | unknown [Medicago truncatula] | 1021 | 6 | 51.78999901 | 35.71000099 | 35.71000099 | 4 | 1.056818008 | 0.646253228 | 0.787045777 | 0.937921524 | 0.100000001 | 0.03604247 |
| gi|356523620 | PREDICTED: 3-oxoacyl-[acyl-carrier-protein] synthase I, chloroplastic-like [Glycine max] | 1030 | 5.96 | 21.17999941 | 8.146999776 | 8.146999776 | 3 | 6.251727104 | 0.02630865 | 6.194410801 | 0.029842161 | 6.309574127 | 0.02505572 |
| gi|356518971 | PREDICTED: pantothenate kinase 2-like [Glycine max] | 1036 | 5.91 | 21.05000019 | 4.933999851 | 4.933999851 | 3 | 0.642687678 | 0.074183248 | 0.711213529 | 0.138720095 | 0.613762021 | 0.045944151 |
| gi|356523918 | PREDICTED: agglutinin-2-like [Glycine max] | 1054 | 5.82 | 31.67000115 | 16.73000008 | 10.67999974 | 5 | 9.727472305 | 0.001320273 | 9.817479134 | 0.00138214 | 10 | 0.001343323 |
| gi|48927683 | putative inorganic pyrophosphatase [Arachis hypogaea] | 1085 | 5.6 | 31.94000125 | 15.27999938 | 15.27999938 | 3 | 0.239883304 | 0.0293351 | 0.580764413 | 0.1447016 | 0.478630096 | 0.087571427 |
| gi|257688087 | unnamed protein product [Glycine max] | 1087 | 5.58 | 64.07999992 | 51.45999789 | 51.45999789 | 14 | 7.447319984 | 0.038405199 | 9.289664268 | 0.0295095 | 8.472273827 | 0.033310462 |
| gi|68264915 | beta-conglycinin alpha subunit [Glycine max] | 1187 | 5.03 | 21.85000032 | 9.272000194 | 5.463999882 | 3 | 0.207014099 | 0.01232289 | 0.1458814 | 0.005100653 | 0.185353205 | 0.009294309 |
| gi|357485127 | Histone H3 [Medicago truncatula] | 1244 | 4.72 | 28.70000005 | 16.14000052 | 10.31000018 | 5 | 2.703958035 | 0.009792863 | 2.654606104 | 0.007649562 | 2.511885881 | 0.005888399 |
| gi|393396088 | ribulose 1,5-bisphosphate carboxylase/oxygenase large subunit (chloroplast) [Vigna unguiculata] | 1286 | 4.55 | 62.11000085 | 26.94999874 | 17.26000011 | 14 | 0.469894111 | 0.024598749 | 0.416869402 | 0.016104961 | 0.469894111 | 0.020957479 |
| gi|388507182 | unknown [Lotus japonicus] | 1692 | 3.9 | 36.59999967 | 10.45999974 | 5.882000178 | 2 | 1.367728949 | 0.03310968 | 1.127197981 | 0.053213779 | 1.116863012 | 0.828370571 |
| gi|356545743 | PREDICTED: myosin-Vb-like [Glycine max] | 1755 | 3.62 | 23.7499997 | 2.896000072 | 1.802000031 | 2 | 0.839460015 | 0.34648031 | 0.744732022 | 0.015870409 | 0.772680581 | 0.032669641 |
| gi|8134607 | RecName: Full=Phosphoglycerate kinase >gi|3319309|gb|AAD09406.1| 3-phosphoglycerate kinase [Funneliformis mosseae] >gi | 1819 | 3.37 | 20.66999972 | 8.894000202 | 6.25 | 2 | 30.19952011 | 0.000521098 | 30.19952011 | 0.000519296 | 29.92264938 | 0.000551217 |
| gi|38146200 | glutamine synthetase [Funneliformis mosseae] >gi | 1855 | 3.21 | 33.89999866 | 11.2999998 | 2.824999951 | 1 | 12.94196033 | 0.044310231 | 10.76465034 | 0.104516603 | 8.790225029 | 0.303579301 |
| gi|356566145 | PREDICTED: L-type lectin-domain containing receptor kinase IX.1-like [Glycine max] | 1892 | 3.1 | 18.26000065 | 6.627000123 | 2.945999987 | 2 | 1.08642602 | 0.006462324 | 1.258924961 | 0.002439791 | 1.127197981 | 0.005538112 |
| gi|163914235 | subtilase [Lotus japonicus] | 1913 | 3.02 | 17.26000011 | 3.953000158 | 1.975999959 | 2 | 2.728977919 | 0.001881571 | 2.630268097 | 0.001863662 | 2.582259893 | 0.001894416 |
| gi|356522113 | PREDICTED: cullin-associated NEDD8-dissociated protein 1-like [Glycine max] | 1921 | 3 | 22.16999978 | 5.17200008 | 1.396000013 | 1 | 0.319153786 | 0.043293089 | 0.654636085 | 0.165531799 | 0.539510608 | 0.092181347 |
| gi|356549592 | PREDICTED: caffeic acid 3-O-methyltransferase-like [Glycine max] | 1989 | 2.79 | 27.88999975 | 7.605999708 | 3.661999851 | 1 | 2.937649965 | 0.0379209 | 3.767038107 | 0.03646446 | 3.908409119 | 0.03650206 |
| gi|502180482 | PREDICTED: abscisic acid 8'-hydroxylase 3-like [Cicer arietinum] | 2163 | 2.42 | 18.65999997 | 6.708999723 | 3.144999966 | 1 | 13.80383968 | 0.03290439 | 11.80321026 | 0.041496329 | 13.80383968 | 0.033945281 |
| gi|356528667 | PREDICTED: uncharacterized protein LOC100799118 [Glycine max] | 2244 | 2.27 | 13.89999986 | 3.133000061 | 1.847000048 | 2 | 1.180320978 | 0.022603029 | 1.270573974 | 0.024498289 | 1.19124198 | 0.031222191 |
| gi|76780890 | binding protein [Rhizophagus intraradices] | 2280 | 2.21 | 36.98000014 | 26.03999972 | 20.37999928 | 12 | 16.90440941 | 0.01311285 | 19.0546093 | 0.01126948 | 20.70141029 | 0.01097528 |
| gi|356496064 | PREDICTED: delta-1-pyrroline-5-carboxylate synthase-like [Glycine max] | 2283 | 2.21 | 30.46000004 | 7.417000085 | 3.443999961 | 2 | 0.01393157 | 0.045931861 | 0.07177943 | 0.088622823 | 0.134276494 | 0.110464603 |
| gi|356552735 | PREDICTED: isocitrate dehydrogenase [NAD] regulatory subunit 1, mitochondrial-like [Glycine max] | 2319 | 2.16 | 24.04000014 | 18.31 | 7.103999704 | 3 | 2.466038942 | 0.028800109 | 2.147830009 | 0.032688431 | 1.67494297 | 0.03313994 |
| gi|356520768 | PREDICTED: stellacyanin-like [Glycine max] | 2424 | 2.08 | 14.04999942 | 9.188999981 | 5.94599992 | 2 | 0.642687678 | 0.070596248 | 0.570164323 | 0.04298152 | 0.242102906 | 0.04298152 |
| gi|388515653 | unknown [Lotus japonicus] | 2464 | 2.07 | 20.74999958 | 5.660000071 | 5.660000071 | 1 | 0.660693526 | 0.0229572 | 0.879022479 | 0.302728713 | 0.751622915 | 0.057563629 |
| gi|356520513 | PREDICTED: protein CASP-like [Glycine max] | 2475 | 2.06 | 17.67999977 | 2.463999949 | 1.593999937 | 1 | 0.642687678 | 0.147834197 | 0.452897608 | 0.044423699 | 0.724435985 | 0.075821541 |
| gi|388511419 | unknown [Lotus japonicus] | 2652 | 2.02 | 18.07000041 | 2.410000004 | 2.410000004 | 1 | 0.01306171 | 0.03040502 | 0.01380384 | 0.03040502 | 0.01037528 | 0.03040502 |
| gi|290766491 | beta-form rubisco activase [Glycine max] | 2709 | 2.01 | 23.01999927 | 2.483000048 | 2.483000048 | 1 | 0.044874541 | 0.051704582 | 0.144544005 | 0.135109305 | 0.02333458 | 0.043216702 |

**Supplementary dataset 2**

| Accession | Description | Index | Unused | %Cov | %Cov(50) | %Cov(95) | Peptides(95%) | GM1/CK2 | PVal GM1/CK2 | GM2/CK2 | PVal GM2/CK2 | GM3/CK2 | PVal GM3/CK2 |
| --- | --- | --- | --- | --- | --- | --- | --- | --- | --- | --- | --- | --- | --- |
| gi|17026394 | UDP-glucose pyrophosphorylase [Amorpha fruticosa] | 10 | 51.9 | 73.25000167 | 62.00000048 | 61.77999973 | 48 | 0.4487454 | 0.01100141 | 0.496592313 | 0.064054631 | 0.461317599 | 0.017318441 |
| gi|372450305 | ATPase subunit 1 (mitochondrion) [Lotus japonicus] | 22 | 41.35 | 60.19999981 | 48.32000136 | 45.35000026 | 35 | 0.401790798 | 0.02144869 | 0.304789513 | 0.000562272 | 0.216770396 | 0.005155133 |
| gi|31580855 | plasma membrane H+-ATPase [Sesbania rostrata] | 26 | 38.99 | 41.42000079 | 25.94000101 | 23.54000062 | 22 | 0.794328213 | 0.633412719 | 0.787045777 | 0.43470791 | 0.416869402 | 0.048959952 |
| gi|257726659 | unnamed protein product [Glycine max] | 35 | 33.79 | 58.60000253 | 47.94000089 | 40.18999934 | 20 | 0.487528503 | 0.007360965 | 0.704693079 | 0.133223906 | 0.591561615 | 0.043891221 |
| gi|356547438 | PREDICTED: pre-mRNA-processing-splicing factor 8-like [Glycine max] | 41 | 30.6 | 25.94999969 | 8.651000261 | 6.955000013 | 15 | 0.654636085 | 0.577459097 | 0.660693526 | 0.02417103 | 0.990831971 | 0.312958986 |
| gi|148872938 | ATP citrate lyase alpha subunit [Glycyrrhiza uralensis] | 45 | 29.23 | 57.73000121 | 37.34000027 | 31.25 | 16 | 0.420726597 | 0.032375991 | 0.672976673 | 0.114886798 | 0.597035289 | 0.083382457 |
| gi|359806735 | uncharacterized protein LOC100812783 [Glycine max] | 51 | 28.24 | 66.99000001 | 44.49999928 | 42.34000146 | 16 | 0.416869402 | 0.007038898 | 0.505824685 | 0.01468059 | 0.529663384 | 0.027977079 |
| gi|357474441 | 26S proteasome non-ATPase regulatory subunit [Medicago truncatula] | 60 | 26.42 | 35.89000106 | 21.89999968 | 18.17000061 | 15 | 0.597035289 | 0.009160244 | 0.608134985 | 0.024296939 | 0.654636085 | 0.0254458 |
| gi|502090101 | PREDICTED: T-complex protein 1 subunit zeta-like [Cicer arietinum] | 72 | 24.19 | 44.11000013 | 34.38999951 | 29.71999943 | 14 | 0.519995987 | 0.009961903 | 0.496592313 | 0.03742462 | 0.452897608 | 0.04151072 |
| gi|255629938 | unknown [Glycine max] | 75 | 23.95 | 64.41000104 | 40.09000063 | 36.93999946 | 14 | 3.80189395 | 9.32E-06 | 3.66437602 | 1.89E-05 | 3.732501984 | 9.72E-06 |
| gi|257742399 | unnamed protein product [Glycine max] | 79 | 23.74 | 57.26000071 | 44.11000013 | 44.11000013 | 20 | 0.672976673 | 0.019898759 | 0.724435985 | 0.115438499 | 0.672976673 | 0.054559682 |
| gi|502134148 | PREDICTED: 5-methyltetrahydropteroyltriglutamate--homocysteine methyltransferase-like [Cicer arietinum] | 80 | 23.62 | 55.21000028 | 37.83999979 | 30.77000082 | 37 | 0.363078088 | 0.168816 | 0.515228629 | 0.01693305 | 0.319153786 | 0.016576881 |
| gi|356521795 | PREDICTED: dihydrolipoyl dehydrogenase-like [Glycine max] | 97 | 22.15 | 42.60999858 | 27.34000087 | 25.44000149 | 13 | 2.91071701 | 5.60E-06 | 3.372873068 | 1.08E-06 | 3.162277937 | 4.01E-06 |
| gi|356513012 | PREDICTED: T-complex protein 1 subunit delta-like isoform 2 [Glycine max] | 101 | 21.87 | 47.47000039 | 30.77000082 | 25.33000112 | 13 | 0.340408206 | 0.044517461 | 0.539510608 | 0.125270799 | 0.380189389 | 0.007770294 |
| gi|356551144 | PREDICTED: alpha-1,4 glucan phosphorylase L isozyme, chloroplastic/amyloplastic-like [Glycine max] | 102 | 21.86 | 37.00999916 | 18.29999983 | 13.60000074 | 12 | 0.465586096 | 0.217102706 | 0.672976673 | 0.120815299 | 0.366437614 | 0.034756009 |
| gi|291047846 | unnamed protein product [Glycine max] | 105 | 21.55 | 48.87999892 | 29.48000133 | 21.0800007 | 12 | 0.685488224 | 0.01193053 | 0.654636085 | 0.043510102 | 0.724435985 | 0.006987272 |
| gi|388508100 | unknown [Lotus japonicus] | 114 | 21.23 | 69.5299983 | 65.67000151 | 57.08000064 | 19 | 3.80189395 | 0.003449852 | 2.606153011 | 0.02560387 | 3.019952059 | 0.008813458 |
| gi|357453895 | 4-hydroxy-3-methylbut-2-en-1-yl diphosphate synthase [Medicago truncatula] | 126 | 20.24 | 36.77000105 | 19.82000023 | 12.90999949 | 10 | 0.492039502 | 0.029357029 | 0.505824685 | 0.01058097 | 0.440554887 | 0.022946211 |
| gi|356557483 | PREDICTED: carbamoyl-phosphate synthase large chain-like [Glycine max] | 127 | 20.24 | 33.75999928 | 15.42000026 | 9.561000019 | 9 | 0.420726597 | 0.000183632 | 0.679203629 | 0.051970359 | 0.416869402 | 0.008317954 |
| gi|90970323 | heat shock protein 60 [Rhizophagus intraradices] | 134 | 19.71 | 44.24000084 | 26.10000074 | 22.54000008 | 11 | 15.70363045 | 0.034615468 | 17.3780098 | 0.026795549 | 17.53881073 | 0.02893455 |
| gi|502098144 | PREDICTED: T-complex protein 1 subunit eta-like [Cicer arietinum] | 146 | 18.91 | 48.3099997 | 19.60999966 | 17.11000055 | 12 | 0.544502676 | 0.033686951 | 0.630957425 | 0.065652572 | 0.55975759 | 0.050542869 |
| gi|356542858 | PREDICTED: beta-amylase-like [Glycine max] | 156 | 18.51 | 31.85000122 | 22.17999995 | 22.17999995 | 21 | 0.444631308 | 0.208313197 | 0.267916799 | 0.0413526 | 0.539510608 | 0.140537098 |
| gi|502115108 | PREDICTED: poly(rC)-binding protein 1-like [Cicer arietinum] | 161 | 18.3 | 28.7800014 | 20.29999942 | 15.12999982 | 9 | 1.644371986 | 0.048316982 | 1.541700006 | 0.072601289 | 1.599557996 | 0.066476583 |
| gi|502144894 | PREDICTED: ubiquitin-NEDD8-like protein RUB1-like [Cicer arietinum] | 178 | 17.67 | 79.22000289 | 61.69000268 | 54.54999804 | 16 | 2.128139019 | 0.099580526 | 2.511885881 | 0.043517862 | 1.923092008 | 0.1809991 |
| gi|356556316 | PREDICTED: probable splicing factor 3A subunit 1-like [Glycine max] | 182 | 17.35 | 33.41999948 | 17.71000028 | 15.20999968 | 11 | 1.819700956 | 0.033231981 | 1.541700006 | 0.098721974 | 1.753880978 | 0.033134099 |
| gi|356553349 | PREDICTED: probable 26S proteasome non-ATPase regulatory subunit 3-like [Glycine max] | 223 | 15.83 | 43.47000122 | 24.07999933 | 18.97999942 | 7 | 0.779830098 | 0.266224712 | 0.515228629 | 0.000849708 | 0.586138189 | 0.009529022 |
| gi|356548123 | PREDICTED: 26S proteasome regulatory subunit 4 homolog A-like [Glycine max] | 242 | 14.89 | 54.18000221 | 31.83000088 | 24.15000051 | 9 | 0.824138105 | 0.59874022 | 0.55462569 | 0.040573571 | 0.698232412 | 0.618034482 |
| gi|502081957 | PREDICTED: UDP-glucuronic acid decarboxylase 6-like isoform X3 [Cicer arietinum] | 262 | 14.49 | 50.85999966 | 34.00000036 | 31.43000007 | 12 | 0.717794299 | 0.154106393 | 0.672976673 | 0.026761111 | 0.515228629 | 0.03225201 |
| gi|300633911 | unnamed protein product [Amorpha fruticosa] | 265 | 14.39 | 44.44000125 | 22.95999974 | 19.63 | 8 | 0.444631308 | 0.007699977 | 0.474242002 | 0.0108805 | 0.478630096 | 0.044941749 |
| gi|54039313 | RecName: Full=40S ribosomal protein S13 | 274 | 14.07 | 68.87000203 | 48.33999872 | 35.76000035 | 8 | 1.67494297 | 0.078417152 | 2.128139019 | 0.022980999 | 2.147830009 | 0.01204724 |
| gi|357479669 | Histone H4 [Medicago truncatula] | 276 | 14.04 | 28.61000001 | 15.48999995 | 14.16999996 | 23 | 5.546257019 | 0.009340271 | 5.495409012 | 0.006256372 | 6.668066978 | 0.005247763 |
| gi|356527232 | PREDICTED: alpha-glucan phosphorylase, H isozyme-like [Glycine max] | 326 | 12.98 | 31.31999969 | 16.67000055 | 15.71999937 | 12 | 0.461317599 | 0.099639289 | 0.229086801 | 0.045232341 | 0.390840888 | 0.116292797 |
| gi|502116230 | PREDICTED: N-carbamoyl-L-amino acid hydrolase-like [Cicer arietinum] | 341 | 12.72 | 31.11999929 | 22.75000066 | 18.88000071 | 7 | 1.958845019 | 0.035838459 | 1.659587026 | 0.111747697 | 2.070141077 | 0.066414639 |
| gi|525345100 | 5-methyltetrahydropteroyltriglutamate--homocysteine methyltransferase-like [Cicer arietinum] | 361 | 12.39 | 51.24999881 | 40.63000083 | 35.38999856 | 43 | 0.107646503 | 0.072731063 | 0.260615289 | 0.141561896 | 0.205116197 | 0.0453825 |
| gi|502078783 | PREDICTED: 40S ribosomal protein S5-like [Cicer arietinum] | 373 | 12.12 | 48.3099997 | 20.76999992 | 16.42999947 | 7 | 2.187762022 | 0.008599441 | 1.406048059 | 0.151330993 | 1.940886021 | 0.033035841 |
| gi|357446813 | Somatic embryogenesis receptor-like kinase [Medicago truncatula] | 381 | 11.99 | 21.88999951 | 8.691000193 | 5.940999836 | 5 | 2.167704105 | 0.02703261 | 2.208004951 | 0.027025539 | 2.187762022 | 0.02703136 |
| gi|378404947 | fumarate reductase [Rhizophagus intraradices] | 383 | 11.97 | 38.94000053 | 19.37000006 | 14.2900005 | 6 | 9.638290405 | 0.001206689 | 12.35947037 | 2.78E-05 | 10.76465034 | 0.000258726 |
| gi|357477179 | Glyceraldehyde-3-phosphate dehydrogenase [Medicago truncatula] | 387 | 11.92 | 79.65000272 | 72.26999998 | 63.71999979 | 58 | 0.251188606 | 0.086679459 | 0.496592313 | 0.1087014 | 0.4487454 | 0.03728826 |
| gi|502151454 | PREDICTED: probable sucrose-phosphate synthase-like [Cicer arietinum] | 393 | 11.82 | 29.10999954 | 11.81000024 | 6.333000213 | 7 | 0.519995987 | 0.032037519 | 0.570164323 | 0.03789033 | 0.549540877 | 0.044144671 |
| gi|356576177 | PREDICTED: uncharacterized protein LOC100803655 [Glycine max] | 454 | 10.66 | 33.68999958 | 16.76000059 | 15.50000012 | 7 | 0.474242002 | 0.1111679 | 0.288403213 | 0.03740396 | 0.642687678 | 0.162910596 |
| gi|356571967 | PREDICTED: UDP-sulfoquinovose synthase, chloroplastic-like [Glycine max] | 501 | 10.12 | 37.52999902 | 11.94999963 | 11.94999963 | 5 | 0.580764413 | 0.178903505 | 0.48305881 | 0.050919551 | 0.452897608 | 0.047211129 |
| gi|351726002 | uncharacterized protein LOC100500302 [Glycine max] | 561 | 9.24 | 43.09000075 | 28.18999887 | 28.18999887 | 6 | 2.398833036 | 0.065064467 | 2.582259893 | 0.043155901 | 2.630268097 | 0.042548358 |
| gi|351721919 | uncharacterized protein LOC100527190 [Glycine max] | 563 | 9.21 | 43.20000112 | 22.32999951 | 22.32999951 | 8 | 1.485936046 | 0.072835527 | 1.614359021 | 0.060111079 | 1.853531957 | 0.042433191 |
| gi|52626570 | alpha-tubulin [Glomus diaphanum] | 603 | 8.82 | 42.03000069 | 29.46999967 | 24.63999987 | 9 | 21.8776207 | 0.000562833 | 21.8776207 | 0.000531978 | 21.47830009 | 0.000538487 |
| gi|356536361 | PREDICTED: unknown protein DS12 from 2D-PAGE of leaf, chloroplastic-like [Glycine max] | 631 | 8.49 | 37.09999919 | 19.42999959 | 16.60999954 | 4 | 1.923092008 | 0.207022801 | 1.870682001 | 0.103552803 | 2.108628035 | 0.043251909 |
| gi|356547865 | PREDICTED: serine carboxypeptidase II-3-like [Glycine max] | 656 | 8.25 | 20.76999992 | 14.04999942 | 8.961000293 | 6 | 45.70882034 | 0.041953091 | 44.05548096 | 0.043914631 | 44.87453842 | 0.04565645 |
| gi|502138074 | PREDICTED: tubulin beta-1 chain-like [Cicer arietinum] | 678 | 8.09 | 61.43000126 | 48.6499995 | 38.33999932 | 27 | 9.727472305 | 0.004973605 | 8.790225029 | 0.009548036 | 9.638290405 | 0.006859026 |
| gi|82792162 | elongation factor 1-alpha, partial [Scutellospora heterogama] | 694 | 8.03 | 46.59999907 | 33.25000107 | 18.19999963 | 14 | 5.70164299 | 0.01076677 | 5.915616989 | 0.009609716 | 5.597576141 | 0.01016092 |
| gi|254212205 | F-ATPase beta subunit, partial (mitochondrion) [Glomus custos] | 695 | 8.03 | 66.18000269 | 55.55999875 | 41.54999852 | 13 | 14.58813953 | 0.01518245 | 16.29295921 | 0.015749549 | 16.90440941 | 0.0130486 |
| gi|84514155 | cytochrome P450 monooxygenase CYP98A37 [Medicago truncatula] | 850 | 6.66 | 29.67000008 | 8.055000007 | 6.876000017 | 3 | 0.48305881 | 0.132359296 | 0.194088593 | 0.01452033 | 0.487528503 | 0.087739579 |
| gi|502134043 | PREDICTED: neutral ceramidase-like [Cicer arietinum] | 917 | 6.29 | 25.87000132 | 9.249000251 | 5.897999927 | 3 | 7.17794323 | 0.02840816 | 8.472273827 | 0.027593561 | 5.248075008 | 0.039108891 |
| gi|502131944 | PREDICTED: carotenoid 9,10(9',10')-cleavage dioxygenase 1-like isoform X1 [Cicer arietinum] | 928 | 6.23 | 25.24999976 | 7.965999842 | 6.949000061 | 6 | 0.325087309 | 0.071015529 | 0.087902263 | 0.00988231 | 0.081658237 | 0.03974263 |
| gi|502169639 | PREDICTED: annexin-like protein RJ4-like [Cicer arietinum] | 970 | 6.05 | 30.79000115 | 11.11000031 | 11.11000031 | 3 | 1.770109057 | 0.049565159 | 1.599557996 | 0.06530717 | 1.485936046 | 0.082694627 |
| gi|356523620 | PREDICTED: 3-oxoacyl-[acyl-carrier-protein] synthase I, chloroplastic-like [Glycine max] | 1030 | 5.96 | 21.17999941 | 8.146999776 | 8.146999776 | 3 | 4.130475044 | 0.04687307 | 4.092607021 | 0.055390731 | 4.168694019 | 0.04397089 |
| gi|356523918 | PREDICTED: agglutinin-2-like [Glycine max] | 1054 | 5.82 | 31.67000115 | 16.73000008 | 10.67999974 | 5 | 13.06171036 | 0.001761993 | 12.94196033 | 0.001857862 | 13.80383968 | 0.001797592 |
| gi|388498786 | unknown [Lotus japonicus] | 1068 | 5.73 | 26.3500005 | 11.65999994 | 7.991000265 | 4 | 3.34194994 | 0.0243554 | 3.40408206 | 0.023604371 | 2.85758996 | 0.034641251 |
| gi|48927683 | putative inorganic pyrophosphatase [Arachis hypogaea] | 1085 | 5.6 | 31.94000125 | 15.27999938 | 15.27999938 | 3 | 0.226986498 | 0.049589131 | 0.586138189 | 0.2051135 | 0.48305881 | 0.1202733 |
| gi|310704426 | stearoyl-acyl carrier protein desaturase [Phaseolus lunatus] | 1086 | 5.6 | 28.3100009 | 17.57999957 | 12.55999953 | 2 | 0.895364821 | 0.211671397 | 0.731139123 | 0.358067304 | 0.597035289 | 0.045083839 |
| gi|257688087 | unnamed protein product [Glycine max] | 1087 | 5.58 | 64.07999992 | 51.45999789 | 51.45999789 | 14 | 7.17794323 | 0.035603069 | 8.629785538 | 0.02759753 | 7.870458126 | 0.0310304 |
| gi|502098976 | PREDICTED: small nuclear ribonucleoprotein-associated protein B'-like isoform X2 [Cicer arietinum] | 1091 | 5.54 | 40.56999981 | 19.57000047 | 14.58999962 | 5 | 2.167704105 | 0.049782261 | 1.923092008 | 0.043871451 | 2.147830009 | 0.081589863 |
| gi|356509826 | PREDICTED: thromboxane-A synthase-like [Glycine max] | 1160 | 5.16 | 17.33999997 | 6.752000004 | 3.832000121 | 2 | 0.847227395 | 0.1168423 | 0.779830098 | 0.026307561 | 0.794328213 | 0.01266292 |
| gi|356540970 | PREDICTED: serine carboxypeptidase-like 34-like [Glycine max] | 1186 | 5.03 | 23.58999997 | 10.01999974 | 2.923000045 | 2 | 2.654606104 | 0.172390401 | 2.779712915 | 0.017451631 | 2.83139205 | 0.02168908 |
| gi|68264915 | beta-conglycinin alpha subunit [Glycine max] | 1187 | 5.03 | 21.85000032 | 9.272000194 | 5.463999882 | 3 | 0.277971298 | 0.026160549 | 0.195884496 | 0.034159239 | 0.248885706 | 0.048531409 |
| gi|357485127 | Histone H3 [Medicago truncatula] | 1244 | 4.72 | 28.70000005 | 16.14000052 | 10.31000018 | 5 | 2.964831114 | 0.01010393 | 2.91071701 | 0.007883275 | 2.779712915 | 0.006060818 |
| gi|393396088 | ribulose 1,5-bisphosphate carboxylase/oxygenase large subunit (chloroplast) [Vigna unguiculata] | 1286 | 4.55 | 62.11000085 | 26.94999874 | 17.26000011 | 14 | 0.524807513 | 0.02639295 | 0.461317599 | 0.017052369 | 0.519995987 | 0.02236687 |
| gi|502168167 | PREDICTED: methionine aminopeptidase 1A-like [Cicer arietinum] | 1462 | 4.07 | 24.94000047 | 8.563999832 | 8.563999832 | 2 | 1.047129035 | 0.001118566 | 1.028015971 | 0.00116104 | 1.202263951 | 0.000953697 |
| gi|359807323 | uncharacterized protein LOC100814078 [Glycine max] | 1562 | 4 | 55.50000072 | 42.78999865 | 40.09999931 | 21 | 38.01893997 | 0.030849921 | 44.87453842 | 0.032941919 | 43.25138092 | 0.03086023 |
| gi|8134607 | RecName: Full=Phosphoglycerate kinase >gi|3319309|gb|AAD09406.1| 3-phosphoglycerate kinase [Funneliformis mosseae] >gi | 1819 | 3.37 | 20.66999972 | 8.894000202 | 6.25 | 2 | 27.28977966 | 0.000543829 | 28.84030914 | 0.000541908 | 28.84030914 | 0.000575969 |
| gi|38146200 | glutamine synthetase [Funneliformis mosseae] >gi | 1855 | 3.21 | 33.89999866 | 11.2999998 | 2.824999951 | 1 | 19.0546093 | 0.061440699 | 18.87990952 | 0.024941109 | 19.95261955 | 0.007866854 |
| gi|502104808 | PREDICTED: uncharacterized protein LOC101505309 [Cicer arietinum] | 1866 | 3.17 | 16.71999991 | 1.785999909 | 0.842600036 | 2 | 2.22843504 | 0.419310004 | 1.786488056 | 0.381390512 | 3.80189395 | 0.044166159 |
| gi|163914235 | subtilase [Lotus japonicus] | 1913 | 3.02 | 17.26000011 | 3.953000158 | 1.975999959 | 2 | 2.53512907 | 0.018231049 | 2.443430901 | 0.01770521 | 2.398833036 | 0.018617719 |
| gi|502180482 | PREDICTED: abscisic acid 8'-hydroxylase 3-like [Cicer arietinum] | 2163 | 2.42 | 18.65999997 | 6.708999723 | 3.144999966 | 1 | 6.08134985 | 0.04594779 | 4.786301136 | 0.060409199 | 5.861382008 | 0.047654741 |
| gi|357438243 | Germin-like protein subfamily 1 member [Medicago truncatula] | 2172 | 2.4 | 15.79000056 | 10.05000025 | 7.176999748 | 1 | 11.37627029 | 0.02960428 | 16.44371986 | 0.027356111 | 6.546361923 | 0.03115657 |
| gi|76780890 | binding protein [Rhizophagus intraradices] | 2280 | 2.21 | 36.98000014 | 26.03999972 | 20.37999928 | 12 | 16.44371986 | 0.01199884 | 19.40885925 | 0.01037662 | 21.28138924 | 0.0101163 |
| gi|356496064 | PREDICTED: delta-1-pyrroline-5-carboxylate synthase-like [Glycine max] | 2283 | 2.21 | 30.46000004 | 7.417000085 | 3.443999961 | 2 | 0.01472312 | 0.04846948 | 0.097274721 | 0.098521948 | 0.177010894 | 0.126202404 |
| gi|502118474 | PREDICTED: chorismate synthase, chloroplastic-like isoform X1 [Cicer arietinum] | 2298 | 2.19 | 37.00999916 | 22.52999991 | 18.8500002 | 7 | 1.599557996 | 0.036001209 | 2.167704105 | 0.034124538 | 1.458814025 | 0.036761489 |
| gi|356514109 | PREDICTED: subtilisin-like protease SDD1-like [Glycine max] | 2405 | 2.09 | 15.90999961 | 2.941000089 | 1.202999987 | 1 | 1.318256974 | 0.025423929 | 1.30617094 | 0.025293 | 1.445440054 | 0.024560969 |
| gi|388515653 | unknown [Lotus japonicus] | 2464 | 2.07 | 20.74999958 | 5.660000071 | 5.660000071 | 1 | 0.619441092 | 0.01913641 | 0.824138105 | 0.181531101 | 0.704693079 | 0.04371148 |
| gi|356520513 | PREDICTED: protein CASP-like [Glycine max] | 2475 | 2.06 | 17.67999977 | 2.463999949 | 1.593999937 | 1 | 1.330453992 | 0.0189365 | 0.928966403 | 1 | 1.472311974 | 0.017962789 |
| gi|388511419 | unknown [Lotus japonicus] | 2652 | 2.02 | 18.07000041 | 2.410000004 | 2.410000004 | 1 | 0.01342765 | 0.03006608 | 0.01419058 | 0.03006608 | 0.01037528 | 0.03006608 |
| gi|290766491 | beta-form rubisco activase [Glycine max] | 2709 | 2.01 | 23.01999927 | 2.483000048 | 2.483000048 | 1 | 0.041686941 | 0.048756178 | 0.115877703 | 0.116845898 | 0.022080051 | 0.04113619 |
